# Tetradecyl 2,3-Dihydroxybenzoate Improves the Symptoms of Diabetic Mice by Modulation of Insulin and Adiponectin Signaling Pathways

**DOI:** 10.3389/fphar.2017.00806

**Published:** 2017-11-13

**Authors:** Lan Xiang, Jing Li, Yanhui Wang, Ruiqi Tang, Qian Wang, Qiaobei Wu, Jianhua Qi

**Affiliations:** College of Pharmaceutical Sciences, Zhejiang University, Hangzhou, China

**Keywords:** insulin, adiponectin, leptin, diabetes, IGF-1

## Abstract

**Background:** Tetradecyl 2,3-dihydroxybenzoate (ABG-001) derived from Chinese medicine, *gentiana regescens* Franch is a leading compound with NGF mimic effect, it can induce neurite outgrowth of PC12 cells via the IGF-1/PI3K/ERK signaling pathway. Thus, we inferred that this compound had anti-diabetic effect and used streptozocin (STZ)-induced diabetic mice to indicate it.

**Methods:** ABG-001 was synthesized with 2,3-dihydroxybenzoic acid and tetradecyl alcohol under certain reaction conditions. STZ-induced diabetic mice were used to investigate anti-diabetic effect. Oral glucose tolerance test, insulin tolerance test, RT-PCR, Western blot, ELISA assays and histological section were performed to do the analysis of action mechanism.

**Results:** ABG-001 showed anti-diabetic effect in STZ-induced diabetic mice. In diabetic mice, the anti-diabetic effect of ABG-001 at a dose of 20 mg/kg was equal with metformin at a dose of 140 mg/kg. Moreover, glucose tolerance and insulin sensitivity were significantly improved on diabetic mice. The plasma insulin, adiponectin and leptin were notably increased, whereas glucagon remarkably decreased. The gene expressions of adiponectin and leptin in adipose tissue, glucose transporter 4 and adiponectin receptor 1 in liver and *gastrocnemius*, ADR2 in hypothalamus and pancreas were obviously increased.

**Conclusion:** ABG-001 exerts antidiabetic effects via modulation of insulin and adiponectin signaling pathways. This new type of molecule could be a promising drug candidate for treatment of diabetes.

## Introduction

Diabetes is mainly characterized by high blood glucose levels and insulin resistance, and it is one of the diseases that are difficult to treat. More severely, serious complications, such as diabetic cataract and nephropathy, develop if the disease is left untreated (Perim et al., 2015; Wu et al., 2015). To date, most diabetic patients still rely on medicine to control blood glucose which they will have to do for the rest of their lives. The available drugs although effectively correct insulin resistance in peripheral tissues, they have obvious side effects such as digestive anorexia, nausea, abdominal pain, diarrhea of metformin (Dujic et al., 2016) and weight and fat gain, early signs of hypertrophic, cardiomyopathy and hepatotoxicity of rosiglitazone (Hemmeryckx et al., 2013). Development of new anti-diabetes drugs with new action mechanism, which mainly targets the cause of insulin resistance is important for diabetes therapy.

Insulin resistance is an important indicator for diabetic patients (Ndisang et al., 2014). Adiponectin is strongly correlated with the insulin sensitivity of an organism. The decreased expressions of adiponectin, its receptors, and circulation level have been found to contribute to the development of insulin resistance (Yadav et al., 2013; Ding et al., 2015). Some evidences reported that adiponectin improved insulin resistance by decreasing triglyceride levels in the liver and muscle cells (Saltiel, 2001; Yamauchi et al., 2001). Moreover, insulin resistance in mice that lacked adipose tissue was completely reversed by the combined treatment of adiponectin and leptin (Yamauchi et al., 2001). The anti-diabetes drug, thiazolidinedione, can induce the mentioned effects by increasing the level of adiponectin (Lihn et al., 2005). Thus, up-regulations of adiponectin and its receptors are promising strategies for treating insulin resistance and diabetes.

Insulin-like growth factor 1, with the same function as insulin, increases the glucose uptake of tissue, promotes glycogen synthesis, and inhibits glycogenolysis (Rinderknecht and Humbel, 1978). Moreover, the molecule is related to cell differentiation, neuron outgrowth, and neuron protection (Xiang et al., 2011; Akundi et al., 2012; Ge et al., 2015). Thus, IGF-1 can be used to treat diabetes, and it can also affect AD. IGF-1 signaling pathway might be the connecting bridge for diabetes and AD therapy.

Anti-diabetes drugs, such as metformin, sulfonylurea, and TZDs have been applied to AD therapy (Perry and Greig, 2004; Gupta et al., 2011; Alagiakrishnan et al., 2013). Moreover, NGF/TrkA signaling has been reported to be associated with insulin synthesis and secretion (Miyagi et al., 2015). This association gave us an inspiration concerning the drugs for AD therapy which may have considerable effect on diabetes. Searching for drugs that will treat both AD and diabetes has become a novel research in the branch of drug development.

ABG-001 (Supplementary Figure 1a) is a candidate drug for the treatment of dementia, with an NGF-mimic activity and neurogenesis function (Luo et al., 2011). Furthermore, this substance induces the neurite outgrowth of PC12 cells by activating the IGF-1 signaling pathway (Tang et al., 2015). In here, we report the anti-diabetic effect of ABG-001 and its action mechanism of STZ-induced mice.

## Materials and Methods

### Preparation of ABG-001 and Other Drugs

The preparation of ABG-001 was conducted according to our previous study (Luo et al., 2011). Briefly, a certain amount of 2,3-dihydroxybenzoic acid, tetrahydrofuran, and N,N-Dicyclohexylcarbodiimide were mixed at 0°C, and tetradecyl alcohol was added 30 min later. The mixture was stirred for 24 h at room temperature. After the reaction had stopped, the filtrate was evaporated and dissolved in ethyl acetate, and the crude product was obtained. The product was chromatographed on silica gel in an open column and eluted with *n*-hexane/ethyl acetate. Then, HPLC (Elite HPLC, Dalian, China) was used to purify ABG-001. The chemical structure, HPLC analysis spectra, and ^1^HNMR of ABG-001 were presented in Supplementary Figure 1.

Streptozocin and MET were purchased from Sigma (Sigma–Aldrich, St. Louis, MO, United States) and Aladdin (Aladdin Bio-chem. Technology Company, Shanghai, China). ABG-001 and MET were dissolved in soybean oil before oral administration; STZ was dissolved in a citrate buffer.

### Construction of Diabetic Model Mice and Animal Experimental Design

In this study, the Institute for Cancer Research (ICR) mice from Zhejiang Academy of Medical Sciences, Hangzhou, China were purchased. They were divided into one cage per five mice and fed with commercial diet (high-fat diet or normal diet) in a clean room at 23 ± 1°C and 50% humidity with a 12:12 light-dark cycle *ad libitum*, respectively. This study was carried out in strict accordance with the recommendations in the Guide for the Care and Use of Laboratory Animals of the National Institutes of Health. All experiments were approved by the Animal Ethics Committee of Medical School, Zhejiang University (Permit Number: ZJU201610128). All efforts were made to minimize death and suffering of the animals.

Seven-week old male ICR mice were fed with high-fat diet for 5 weeks and were intraperitoneally injected with 40 mg STZ per kilogram of body weight per day after fasting 12 h, continuous injection for 3 days. Meanwhile normal control mice were intraperitoneally injected citrate buffer. After 2 weeks of injection, the fasting glucose of mice was measured. Experiment on diabetic mice model was considered to be successful if the glucose concentration was higher than 12 mmol/L.

Ten normal mice and 50 STZ-induced diabetic mice at 12 weeks age in one trail were randomly divided into six groups: control (normal mice), DM-C, DM plus MET group, and DM plus ABG-001 groups, each group having 10 mice, and oral administration was used. The control groups were treated with vehicle solvent every day. The positive control group received MET at a dose of 140 mg/kg per day. ABG-001-treated groups received ABG-001 at doses of 5, 10, and 20 mg/kg per day, respectively. The experimental period was 3 weeks, and the fasting glucose, body weight, food intake, and water consumption were recorded every week in this period. This experiment was repeated four times. At the end of the experiment, the mice were sacrificed and the blood, heart, liver, pancreas, spleen, kidney, white adipose tissue, *gastrocnemius* muscle, and brain of the mice were weighed and taken as samples. They were stored at -30°C for further analysis.

### Detection of Fasting Glucose and Plasma Biochemical Indexes

At the end of every week, mice were fasted overnight, and the fasting glucose of mice was measured with glucometer (Andon Health, Tianjin, China). The blood was collected from the mice orbit using a capillary tube, centrifuged at 3000 rpm for 15 min, and the supernatant was obtained as serum. In addition, plasma samples of the diabetic mice at 30 min in glucose tolerance test were obtained. Insulin, glucagon, leptin, adiponectin were measured using ELISA Assay Kits (insulin and leptin, Westang Bio-Tech, Shanghai, China; adiponectin, Cusabio, Wuhan, China) in accordance with the manufacturer’s instructions. At the end of experiment, the plasma samples were sent to Hangzhou Dian Medical Test Center (Hangzhou City, Zhejiang Province, China), for the measurement of biochemical indexes using Assay Kits (Roche, Basel, Switzerland).

### Glucose and Insulin Tolerance Tests

In this study, we measured the glucose tolerance of STZ-induced diabetic mice after administrating ABG-001 for one time or 3 weeks. At first, the diabetic mice were fasted 14 h and orally administrated glucose at a dose of 2 g/kg to one mouse. The blood glucose concentrations of mice from tail vein at the indicated times points were measured with glucometer.

Subsequently, insulin tolerance test (ITT) was performed. The STZ-induced diabetic mice were fasted for 4 h. After an intraperitoneal injection of 0.8 units/kg of bovine insulin (Yeason, Shanghai, China), glucose concentrations of the STZ–induced diabetic mice were measured from tail vein at 0, 15, 30, 45, and 60 min.

### Measurement of Glycogen

The glycogen level in the liver or *gastrocnemius* was measured using the Glycogen Assay Kit (Bioengineering Institute of Nanjing Jiancheng Company, Nanjing, China) according to the manufacturer’s instructions. Approximately, 75 mg of liver or *gastrocnemius* was added to alkaline solution with a ratio of 1: 3 and incubated in water bath at 100°C for 20 min. After cooling with flowing water, the reaction mixture of liver or *gastrocnemius* muscle was diluted with distilled water to 1% tracer liquid and the OD was measured at 620 nm wavelength.

### RT-PCR Analysis

RNA extraction and cDNA synthesis of white adipose tissue, liver, *gastrocnemius* muscle, pancreas, and hypothalamus were performed using the methods from previous studies (Tang et al., 2015). The transcript levels were quantified by using CFX96-Touch (Bio-Rad, Hercules, CA, United States) and SYBR Premix EX Taq^TM^ (Takara, Otsu, Japan). The primers (Sangon Tech, Shanghai, China) in this study are displayed in Supplementary Table 1. We amplified cDNA using the following conditions: 95°C for 2 min, followed by 40 cycles for 15 s at 95°C, and 35 s at 60°C. All results were standardized to *18S* RNA or GAPDH gene expression, and relative mRNA transcript levels were reckoned using the DDCt formula.

### Western Blot Analysis

The protein sample of each liver with 200 mg was prepared and protein concentration was measured as described in a previous study (Tang et al., 2015). One hundred microgram protein of each sample was loaded onto the 10% SDS-PAGE gel and run at 120 V for 60 min. Subsequently, the protein on the gel was transferred to polyvinylidenedifluoride membranes. The membrane was blocked with 5% non-fat milk in TBS for 60 min, incubated with first antibodies of IGF-1 receptor, phosphor-IGF-1 receptor, AKT (Cell Signaling Technology, Danvers, MA, United States), phosphor-AKT (Abcam, Hong Kong, China), and GAPDH (Beijing ComWin Biotechnology, Beijing, China) in 2% non-fat milk overnight at 4°C, respectively. Afterward, the membrane was incubated with a secondary antibody (Beijing ComWin Biotechnology, Beijing, China) for 45 min after washing three times with TBS. The bands were developed using enhanced chemiluminescent reaction (Beijing ComWin Biotechnology, Beijing, China), and density analysis was performed using Image J software (National Institute of Health, Rockville, MD, United States).

### Assessment of Histological Sections

The samples of pancreas after administrating ABG-001 at a dose of 20 mg/kg for 0, 2, 4, and 6 weeks were preserved in 10% formalin solution followed by tissue dehydration with alcohol and xylene. Then, each sample was embedded in paraffin wax, sectioned at 5 μm, and mounted on slides prior staining. Hematoxylin and eosin stains were used. The slides were observed under the light microscope, and the observations were recorded using 20× lenses.

### Statistical Analysis

Animal experiments were independently repeated four times, and the data was presented as mean ± SEM. Significant differences between groups were analyzed through One-way ANOVA followed by Tukey post-test of GraphPad Prism software (GraphPad Prism). ^∗^*p* < 0.05 or ^#^*p* < 0.05 represents a statistically significant difference between the two groups.

## Results

### ABG-001 Improves the Clinical Symptoms and Energy Metabolism Disorder of STZ Mice

**FIGURE 1:**
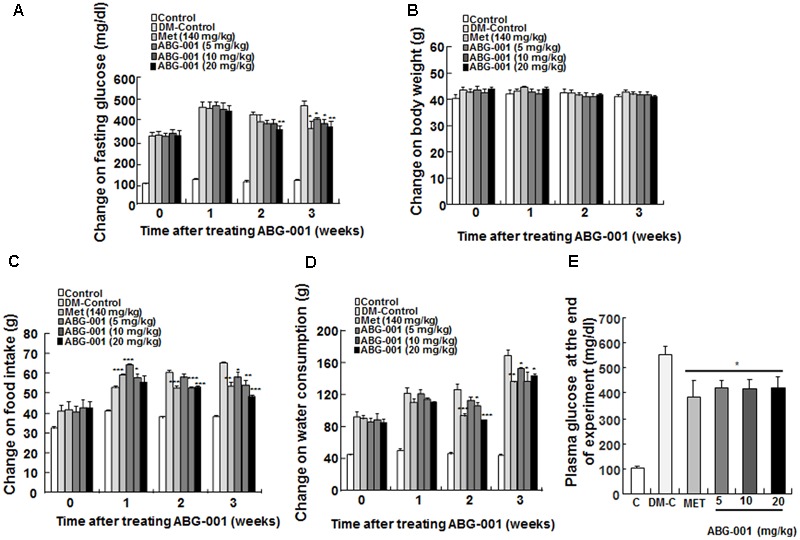
Anti-diabetic effects of ABG-001 on STZ-induced diabetic mice. The changes in fasting glucose **(A)**, body weight **(B)**, food intake **(C)**, water consumption **(D)**, and plasma glucose at end of experiment **(E)** of STZ-induced diabetic mice after administration of ABG-001 and MET. Each value was expressed as the means ± SEM of the 10 mice. ^∗^*p* < 0.05, ^∗∗^*p* < 0.01, and ^∗∗∗^*p* < 0.001 indicate a significant difference compared with the DM-C group.

The clinical symptom changes in STZ-induced diabetic mice after administration of ABG-001 and MET are presented in **Figure 1**. Fasting glucose (**Figure 1A**), food intake (**Figure 1C**), and water consumption (**Figure 1D**) of mice in MET and ABG-001-treated groups were significantly reduced after administrating MET and ABG-001 for 1, 2, and 3 weeks, respectively (*p* < 0.05, *p* < 0.01, and *p* < 0.001). Clear changes in the body weight of the treated group were not observed (**Figure 1B**). In addition, the wetness of animal bedding was significantly attenuated. Meanwhile, the plasma TG and TC of mice in 20 mg/kg ABG-001-treated group were significantly decreased (*p* < 0.05, *p* < 0.05); and plasma HDL, LDL, AST, and ALT showed no difference in comparison with the DM-Control (Supplementary Figures 2, 3). These results indicate that ABG-001 can improve the symptoms of hyperphagia, polydipsia, and hyperglycemia of STZ-induced mice and energy metabolism disorder; and it has no toxic or side effects.

### ABG-001 Improves Glucose and Insulin Tolerance

**FIGURE 2:**
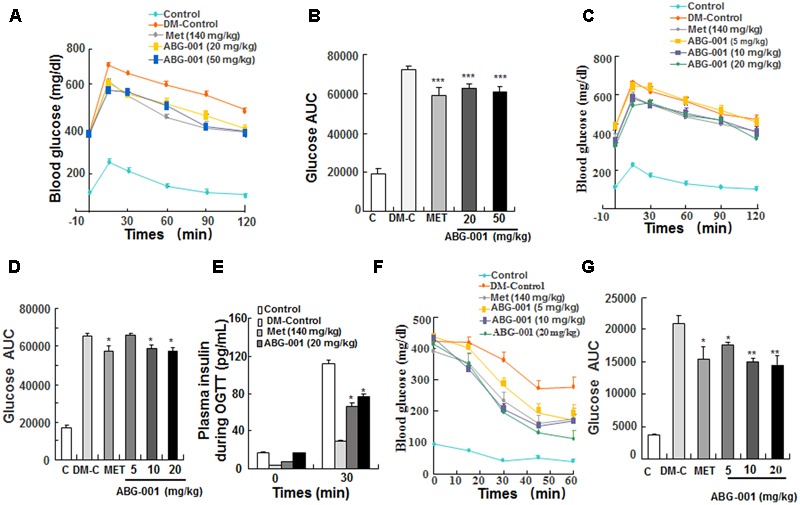
Effects of ABG-001 on tolerance of glucose and insulin in STZ-induced diabetic mice. Changes in fasting glucose and AUC during OGTT in acute administration of ABG-001 **(A,B)** and after administration ABG-001 for 3 weeks **(C,D)**. Change in the plasma insulin during OGTT after administration ABG-001 and metformin for 3 weeks **(E)**. Changes in fasting glucose **(F)** and AUC **(G)** of STZ-induced diabetic mice in ITT after administration ABG-001 for 3 weeks. Each value was expressed as the means ± SEM of five or eight mice. ^∗^*p* < 0.05, ^∗∗^*p* < 0.01, and ^∗∗∗^*p* < 0.001 indicate a significant difference compared with the DM-Control mice group.

The oral glucose tolerance and ITTs were performed in STZ-induced diabetic mice. The glucose tolerance of mice was significantly improved after single oral administration of ABG-001 at doses of 20 and 50 mg/kg and metformin at 140 mg/kg (**Figure 2A**). Meanwhile, the values of area under the curve (AUC) in these groups were significantly lowered comparing with DM-control group (**Figure 2B**, *p* < 0.001). Furthermore, we tested the glucose tolerance of mice after treatment with ABG-001 and metformin for 3 weeks. Interestingly, ABG-001 doses at 10, 20 mg/kg like MET lowered blood glucose levels with a faster onset (**Figure 2C**, *p* < 0.05) and lower AUC (**Figure 2D**, *p* < 0.05). In addition, the plasma insulin levels of 20 mg/kg ABG-001 and metformin groups were significantly increased at 30 min (**Figure 2E**, *p* < 0.05). Next, we injected insulin to the mice and did ITT. The blood glucose levels in 5, 10, 20 mg/kg ABG-001 and metformin treated mice were significantly lowered when compared to that in DM-Control mice (**Figure 2F**), and the AUC in ABG-001 groups was significantly decreased (**Figure 2G**, *p* < 0.05, *p* < 0.01, *p* < 0.01). These results suggest that ABG-001 increases insulin; ameliorates insulin sensitivity and glucose tolerance.

### Effects of ABG-001 on the Genes Expression, IGF-1 Signaling, and Hepatic Glycogen in Liver

**FIGURE 3:**
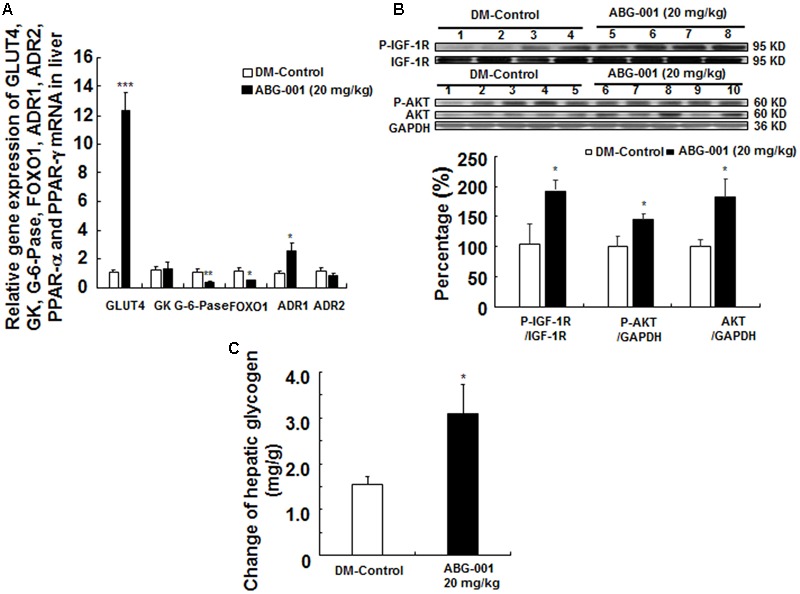
Effects of ABG-001 on the livers of STZ-induced diabetic mice after administrating ABG-001 for 3 weeks. Changes in the gene expressions of GLUT4, GK, G-6-pase, FOXO1, ADR1, ADR2 in the liver after administrating ABG-001 **(A)**. Each value was expressed as the means ± SEM of eight mice. Western blot analysis of liver samples **(B)**. Lanes: 1–4, DM-Control; 5–8, ABG-001 at a dose of 20 mg/kg. Each value was expressed as the means ± SEM of five mice. The change in hepatic glycogen after ABG-001 treatment **(C)**. ^∗^*p* < 0.05, ^∗∗^*p* < 0.01, and ^∗∗∗^*p* < 0.001 indicate a significant difference compared with the DM-C group.

The genes expressions of glucose transport 4, glucose kinase, glucose 6 phosphate kinase, forkhead box O1, ADR1, ADR2 in the liver are shown in **Figure 3A**. ABG-001 increased the mRNA expressions of GLUT4 and ADR1 (*p* < 0.001 and *p* < 0.05), and decreased the mRNA expressions of G-6-pase and FOXO1 (*p* < 0.01 and *p* < 0.05). However, the genes expression of ADR2 was not affected by ABG-001. The protein levels of IGF-1R, protein kinas B (AKT), and their phosphorylation in the liver after treatment with ABG-001 are given in **Figure 3B** and Supplementary Figure 6. ABG-001 significantly increased phosphorylation of IGF-1R, AKT, and AKT protein levels in comparison with diabetic control group (*p* < 0.05, *p* < 0.05, and *p* < 0.05). The change in glycogen in the liver after treatment with ABG-001 is found in **Figure 3C**. The marked increase in hepatic glycogen was observed (*p* < 0.05). These results indicate that ABG-001 decreased plasma glucose via promotion of the genes expressions of GLUT4, ADR1, IGF-1 signaling transmission, and the synthesis of hepatic glycogen.

### Protective Effect of ABG-001 on Pancreas of STZ-Induced Diabetic Mice

**FIGURE 4:**
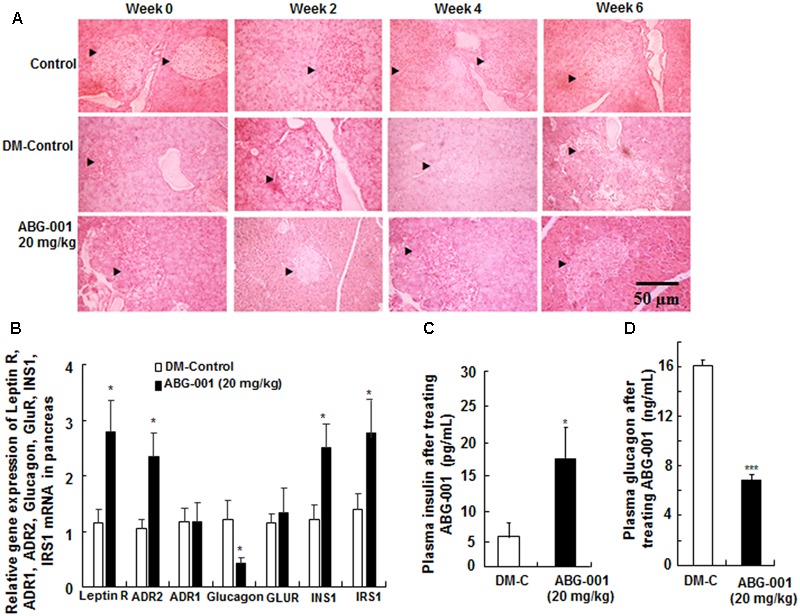
Effects of ABG-001 on the pancreas of STZ-induced diabetic mice. Histological changes in the pancreas after treatment with ABG-001 for 6 weeks **(A)**. Black arrows indicated the changes process of necrotic regions of islets in pancreas after treating ABG-001 at a dose of 20 mg/kg. Changes in the gene expressions of leptin receptor, ADR1, ADR2, glucagon, GLUR, INS1, and IRS1 in pancreas **(B)**. ABG-001 increased plasma insulin **(C)**, but decreased plasma glucagon **(D)** levels at end of experiment. Each value was expressed as the means ± SEM of eight mice. ^∗^*p* < 0.05 and ^∗∗∗^*p* < 0.001 indicate a significant difference compared with the DM-C group.

The histomorphological changes on the pancreas after treatment with ABG-001 for 0, 2, 4, and 6 weeks are shown in **Figure 4A**. The islets of pancreas in DM-control group were destroyed over time; hence, the islet cells and shape could still be observed in the pancreas after administration of ABG-001 at dose of 20 mg/kg. The gene expressions of leptin receptor, ADR1, ADR2, glucagon, GLUR, INS1, and IRS1 of pancreas were shown in **Figure 4B**. The significant increase in leptin receptor, ADR2, INS1 and IRS1 and decrease in glucagon mRNA expressions were observed (*p* < 0.05, *p* < 0.05, and *p* < 0.05, respectively). Meanwhile, plasma insulin of diabetic mice was also increased by ABG-001 (**Figure 4C**, *p* < 0.05), and glucagon in plasma was significantly decreased (**Figure 4D**, *p* < 0.001). These results suggest that ABG-001 can improve the damage of pancreas of diabetic mice induced by STZ.

### Effects of ABG-001 on Epidydimal Adipose Tissue and Hypothalamus of STZ-Induced Diabetic Mice

**FIGURE 5:**
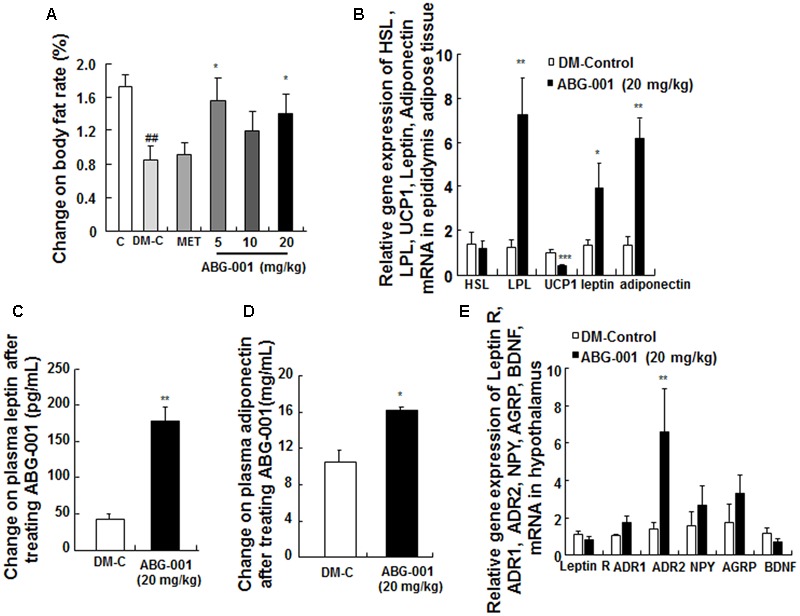
Effects of ABG-001 on epididymal adipose tissue and hypothalamus in STZ-induced diabetic mice. Changes in the genes expressions of HSL, LPL, UCP1, leptin, and adiponectin in epididymal adipose tissue **(A)**, plasma leptin **(B)**, and adiponectin **(C)** after administrating ABG-001 for 3 weeks. Changes in the gene expressions of leptin receptor, ADR1, ADR2, NPY, AgRP, and BDNF in hypothalamus **(D)**. Each value was expressed as the means ± SEM of six or eight mice **(E)**. ^∗^*p* < 0.05 and ^∗∗^*p* < 0.01 indicate a significant difference compared with the DM-C group. ^##^*p* < 0.01 indicate a significant difference compared with the control group.

The change of body fat rate was showed in **Figure 5A**. The changes in the gene expressions of HSL, LPL, UCP1, leptin, and adiponectin in epidydimal adipose tissue are presented in **Figure 5B**. ABG-001 significantly increased the mRNA expression levels of LPL, leptin, and adiponectin in adipose tissue (*p* < 0.05, *p* < 0.05, and *p* < 0.05). Furthermore, the plasma leptin and adiponectin were also obviously higher than that of DM-control group (**Figures 5C,D**, *p* < 0.01, *p* < 0.05). The changes in the gene expressions of leptin receptor, ADR1, ADR2, NPY, AgRP, and BDNF in hypothalamus are shown in **Figure 5E**. The amount of ADR2 mRNA was significantly increased (*p* < 0.05). However, ABG-001 did not affect the other genes expression. These results suggest that ABG-001 improved the energy metabolism disorder and satiated the polyphagia caused by diabetes by regulating adiponectin, leptin and ADR2 gene expressions.

## Discussion

ABG-001 is a leading compound derived from the neuritogenic compound gentisides of Chinese medicine, *gentiana regescen* Franch. The compound contains a long alkyl chain of 14 carbons, two close hydroxyl groups on the benzene ring, and an ester linkage between the ring and alkyl chain (Luo et al., 2011). ABG-001 can induce neurite outgrowth of PC12 cells like NGF via the IGF-1/PI3K/ERK signaling pathway (Tang et al., 2015). In the present study, we tested the anti-diabetic effects of ABG-001 on STZ-induced diabetic mice. The administration of the compound alleviated hyperphagia, polydipsia (**Figures 1C,D**), and hyperglycemia (**Figures 1C,E**), and lipid metabolism disorder caused by diabetes (Supplementary Figure 2). At this point, the result was consistent with the effects of other anti-diabetes drugs (Wang et al., 2014). The anti-diabetic effect of ABG-001 at 20 mg/kg was equivalent to that of MET at 140 mg/kg. However, ABG-001 did not display the dose-dependent relationship in our study. It is reason why ABG-001 only has 8–10% bioavailability. ABG-001 in ingestion at high doses was not able to absorb into blood. Most of the ABG-001 remained in the gut or excreted in the urine and stools.

We used transgenic db/db mice to confirm the anti-diabetic effect of ABG-001. ABG-001 decreased fasting glucose, however, it had no influence on the food intake and water consumption of db/db mice (Supplementary Figure 4). Possibly, the leptin receptor of the db/db mice, which attends to the regulation of food intake in hypothalamus, was knocked out. This result was also observed in the studies of antidiabetes drugs like latanoprost and niclosamide (Wang et al., 2013; Tao et al., 2014).

Liver is an important organ that regulates glycometabolism and energy metabolism. GLUT, GK and G-6-pase, ADR, and FOXO1 play important roles in mediating hepatic glucose production (Nakae et al., 2001; Sharabi et al., 2015). Moreover, increasing IGF-1 in peripheral tissues promotes glucose uptake (Moses et al., 1996) and IGF-1 signaling pathway takes an important role in neuritogenic effect of ABG-001 (Tang et al., 2015). Thus, we explored the changes in these genes and protein expressions in the liver. The increase in GLUT4, ADR1 gene expressions, hepatical glycogen, phosphorylation of IGF-1R and AKT, and the reduction of G-6-pase and FOXO1 gene expression in our study indicate that ABG-001 reduced blood glucose level by increasing glucose transport, hepatical glycogen synthesis, and IGF-1 signaling transmission.

Adipose tissues express and secrete many kinds of adipocytokines, such as adiponectin and leptin, to maintain the homeostasis of body fat and blood glucose (Cao, 2014). These two hormones target their receptors which are located in the hypothalamus to control food intake (Qi et al., 2004). Therefore, increasing adiponectin or improving ADR function had been become important in the research for diabetic therapy. In our study, the increase in adiponectin gene expression in adipose tissue (**Figure 5B**), plasma adiponectin (**Figure 5D**), and ADR1 or ADR2 gene expression in the liver (**Figure 3A**), hypothalamus (**Figure 5E**), pancreas (**Figure 4B**), and gastrocnemius (Supplementary Figure 5a) suggest that adiponectin signaling pathway plays an important role in the anti-diabetic effects of ABG-001.

Decrease in PI3K activity, reduction of GLUT4 transposition, and inhibition of glucose transport are essential factors in inducing insulin resistance in the muscle tissue of diabetic mice (Ji et al., 2014). The increase in GLUT4 and ADR1 gene expressions, and muscle glycogen in muscle tissue after treatment with ABG-001, as shown in Supplementary Figures 5a,b, clarifies that ABG-001 improved insulin resistance of the muscle by enhancing GLUT4 and adiponectin signaling transmission.

In our study, the residual islets that were observed in the pancreas after ABG-001 treatment, as depicted in **Figure 4A**, insulin-related gene expression and plasma insulin were significantly increased. These results suggested that ABG-001 may be an insulin secretagogue, like the sulfonylurea class of anti-diabetic medications such as glipizide. STZ induces the state of hypoinsulinemia and hyperglycemia in mice by destroying the pancreatic β cells due to its nature of preferential toxicity toward pancreatic β cells (Graham et al., 2011). Thus, ABG-001 might potentially act by reversing the toxicity and damage to pancreatic β cells, and contribute to better insulin release profile. Furthermore, leptin has the ability to protect the pancreas against inflammatory damage (Jaworek and Konturek, 2014). The increase in plasma leptin, leptin receptor, and ADR2 gene expressions indicates that ABG-001 protected the pancreas via the leptin receptor and ADR2 pathway.

During experiment, we found that the effects of glucose and ITTs were not consistent with insulin secretion (**Figure 2E**) and insulin sensitivity (**Figure 2F**) along with the almost 12-fold increase in liver and almost fourfold increase in muscle GLUT4 expression (**Figure 3** and Supplementary Figure 5). It is reason why the glucose and ITTs and gene expression measurement were not performed in the same mice.

## Conclusion

**FIGURE 6:**
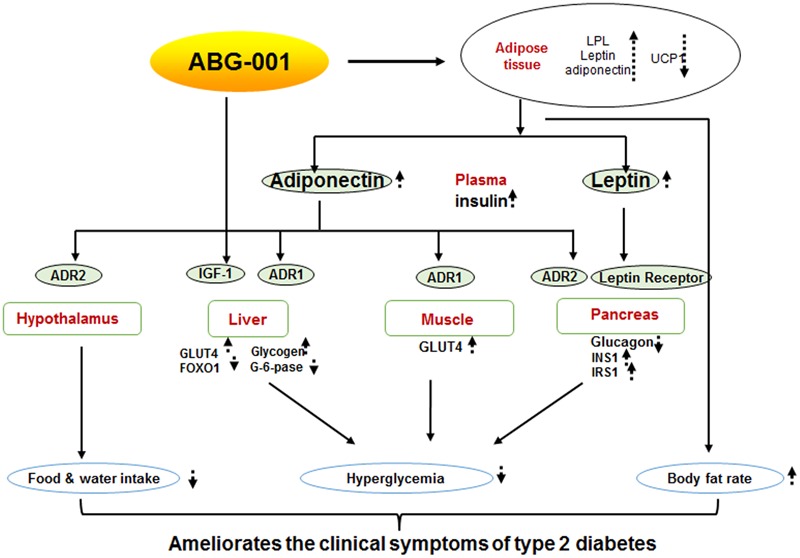
Proposed mechanism of ABG-001. Insulin and adiponectin signaling pathways play important roles in the anti-diabetic effects of ABG-001.

In summary, we report that ABG-001 is a low toxicity candidate drug with anti-diabetic and anti-AD effects. Insulin and adiponectin signaling pathways have important roles in the anti-diabetic effects of ABG-001 (**Figure 6**). This knowledge can be used in developing a drug with anti-AD and anti-diabetic effects in the future.

## Author Contributions

JL, YW, RT, QWa, and QWu conducted the research and analyzed the data; LX guided research and wrote the manuscript; JQ contributed to the experimental design and revising manuscript.

## Conflict of Interest Statement

The authors declare that the research was conducted in the absence of any commercial or financial relationships that could be construed as a potential conflict of interest.

## Abbreviations

AD: Alzheimer’s disease
ADR1: adiponectin receptor 1
ADR2: adiponectin receptor 2
AgRP: agouti-related peptide
ALT: alanine aminotransferase
ARC: activity-regulated cytoskeleton-associated protein
AST: aspartate amino transferase
DM-C: diabetes mellitus-control
FFA: free fatty acid
FOXO1: forkhead box O1
G-6-pase: glucose-6-phosphase
GLP-1: glucagon-like peptide1
GLUT: glucose transporter
HPLC: high performance liquid chromatography
IGF-1: insulin like growth factor 1
INS1: insulin substrate 1
IRS1: insulin receptor substrate 1
LPL: lipoprotein lipase
MET: metformin
NGF: nerve growth factor
NPY: neuropeptide Y
PI3K: phosphoinositide 3-kinases
STZ: streptozocin
TC: total cholesterol
TG: triglyceride
TrkA: tyrosine kinase A
TZDs: thiazolidinediones
UCP1: uncoupling protein 1
