# Supplementary material for: Tetradecyl 2,3-Dihydroxybenzoate Improves the Symptoms of Diabetic Mice by Modulation of Insulin and Adiponectin Signaling Pathways
Source: Front Pharmacol. 2017 Nov 13;8:806. doi: 10.3389/fphar.2017.00806 (PMC5693855; doi:10.3389/fphar.2017.00806)
Supplement: Supplementary file 1 [file Data_Sheet_1.DOCX]

**Tetradecyl 2,3-dihydroxybenzoate improves the symptoms of type-2 diabetic mice by modulation of insulin and adiponectin signaling pathways**

**Lan Xiang, Jing Li, Yanhui Wang, Ruiqi Tang, Qian Wang, Qiaobei Wu, Jianhua Qi ^*^**

College of Pharmaceutical Sciences, Zhejiang University, China;

**Supplementary information**

**(a)**

**(b)**

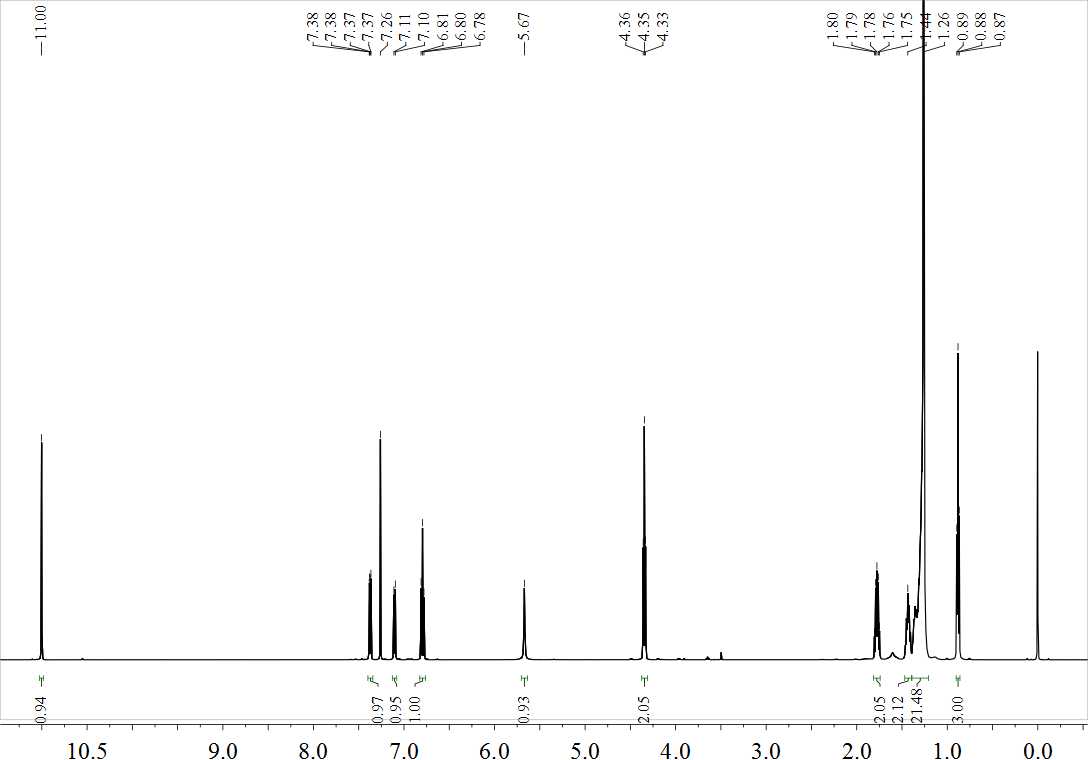
**(c)**

**Supplementary Fig. 1. Purification and structure determination of ABG-001. (a)** Chemical structure of ABG-001. **(b)** The HPLC analysis spectra of ABG-001, condition: 5C18-MS-II; UV 210 nm; 1 mL/min; 94% MeOH, retention time of ABG-001 is 14.54 min. **(c)** ^1^H-NMR spectra of ABG-001.

***
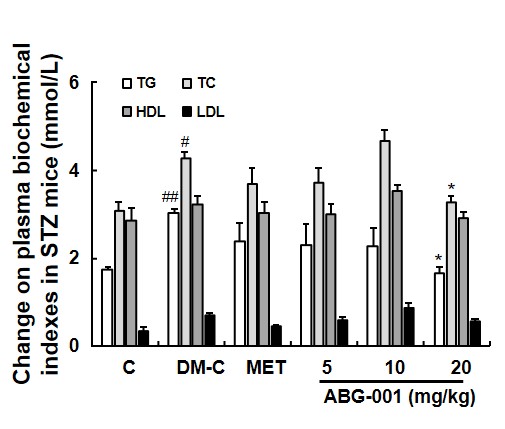
***

**Supplementary Figure 2. ABG-001 ameliorated the lipid metabolism in STZ-induced mice after administrating ABG-001 for three weeks. ^#^** represents significant difference compared with the normal control group at p < 0.05. *p < 0.05 indicated significant difference compared with the DM-control group. Animal number is 7 or 9.

**
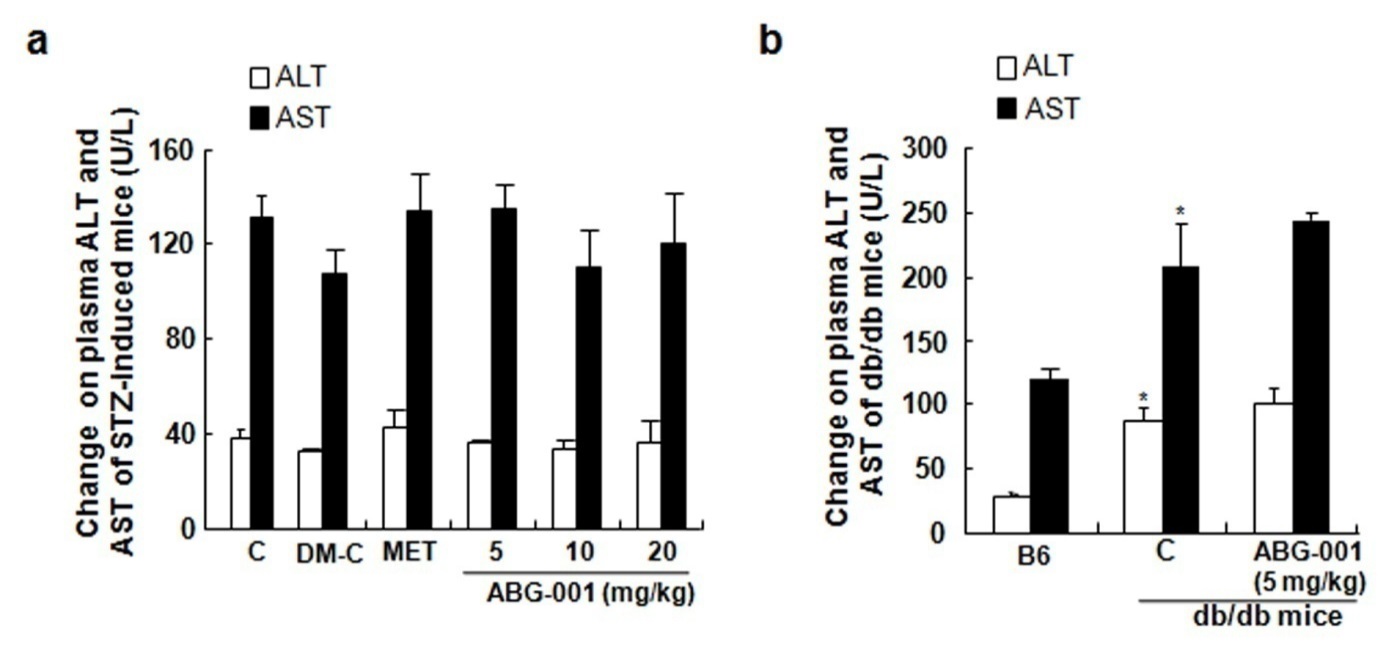
**

**Supplementary Fig. 3. ABG-001 did not affect the plasma ALT and AST of STZ-mice** Changes in the plasma ALT and AST levels in STZ mice. Each value was expressed as the means ± SEM of 7 or 8 mice.


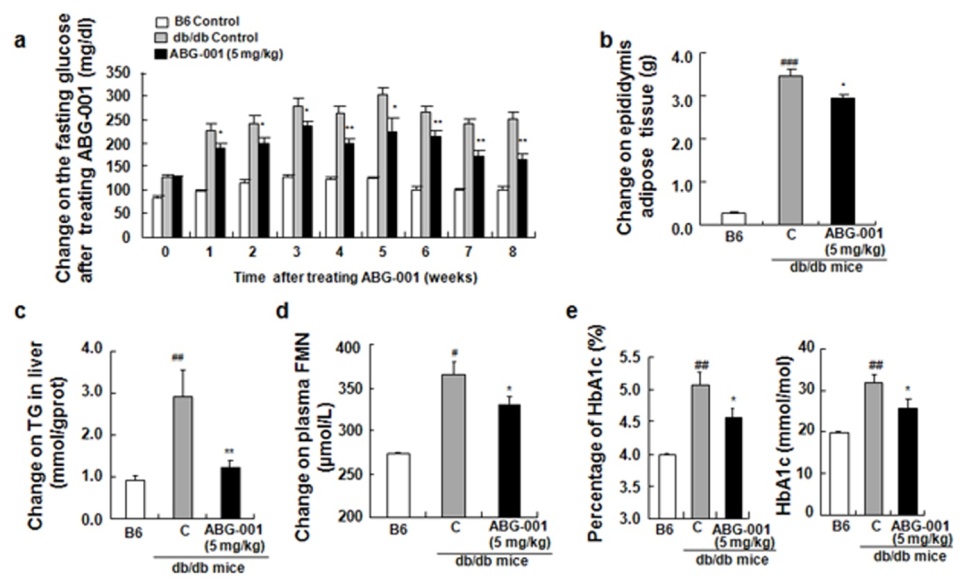


**Supplementary Fig. 4. Antidiabetes effect of ABG-001 on db/db mice.** Changes in fasting glucose (a), weight of epididymis adipose (b) and TG levels in the livers (c) of db/db mice after administrating ABG-001. ABG-001 significantly decreased FMN (d) and HbA1c (e) levels compared with db/db mice. B6 mice were used as normal control, and the experimental period was eight weeks. Each value was expressed as the means ± SEM of 5 or 8 mice. ^#^p < 0.05 and ^##^p < 0.01 indicate a significant difference compared with the B6 mice group. **^*^**p < 0.05 and **^**^**p < 0.01 represent a significant difference compared with the db/db mice group.


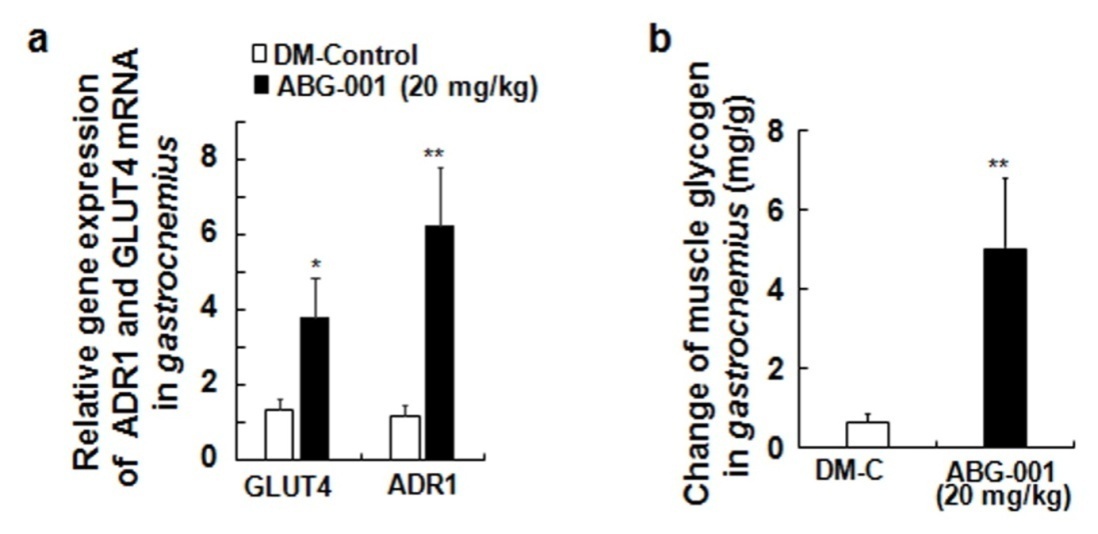


**Supplementary Fig. 5. Effect of ABG-001 on the *gastrocnemius* of STZ-induced diabetic mice.** Changes in the gene expression of GLUT4 and ADR1, and muscle glycogen in *gastrocnemius* of STZ-induced diabetic mice after ABG-001 treatment for three weeks. Each value was expressed as the means ± SEM of 8 mice. ^*, **^ represent a significant difference between DM-control and ABG-001 treated group at p < 0.05 and p < 0.01, respectively.


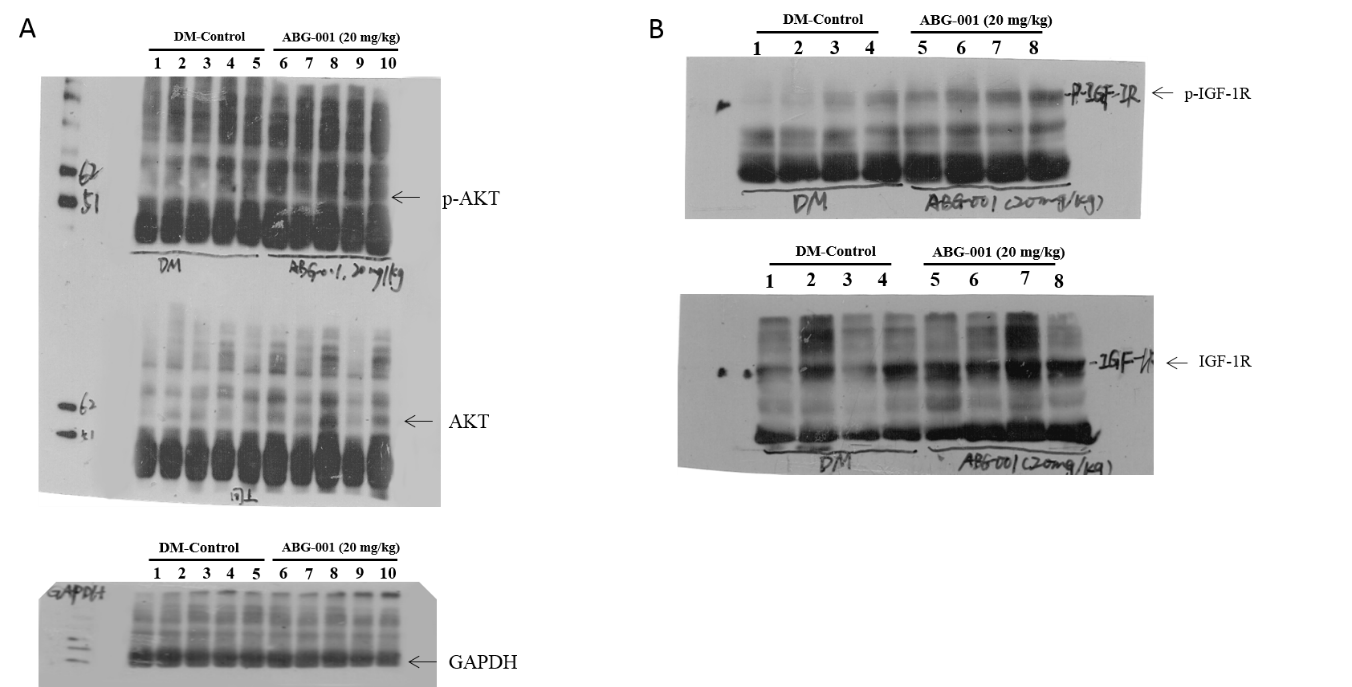


**Supplementary Fig. 6. The original full gels of western blotting analysis.** The protein bands shown in Fig. 3b are indicated by arrows.

**Supplementary Table 1. Primers sequence in RT-PCR analysis**

| **Gene** | **Species** | **Sequences** |
| --- | --- | --- |
| **Adiponectin** | **mouse** | **sense: 5’-TGACGACACCAAAAGGGCTC-3’** |
|  |  | **anti-sense: 5’-ACCTGCACAAGTTCCCTTGG-3’** |
| **ADR1** | **mouse** | **sense: 5’-AGATAGGACGTGGGAGCTCAT-3’** |
|  |  | **anti-sense: 5’-ATGGCCCGTTATCAGCCAG-3’** |
| **ADR2** | **mouse** | **sense: 5’-GGAGATTTGGAGCCCAGCTT-3’** |
|  |  | **anti-sense: 5’-GGCCTTCCCACACCTTACAA-3’** |
| **AGRP** | **mouse** | **sense: 5’-GGCCTCAAGAAGACAACTGC-3’** |
|  |  | **anti-sense: 5’-GACTCGTGCAGCCTTACACA-3’** |
| **BDNF** | **mouse** | **sense: 5’- TTGTTTTGTGCCGTTTACCA-3’** |
|  |  | **anti-sense: 5’-GGTAAGAGAGCCAGCCACTG-3’** |
| **FOXO1** | **mouse** | **sense: 5’-TCGGCTGAATGACTGAACCT-3’** |
|  |  | **anti-sense:5’-GACCTGTACAAAGCTGGCAC-3’** |
| **G-6-Pase** | **mouse** | **sense: 5’-AATCTCCTCTGGGTGGC-3’** |
|  |  | **anti-sense: 5’-GCTGTAGTAGTCGGTGTCC-3’** |
| **GK** | **mouse** | **sense: 5’-CCCGAGAGATTTTGGATGCC-3’** |
|  |  | **anti-sense: 5’-GCAGTTGTTTCAGGCATGGA-3’** |
| **Glucagon** | **mouse** | **sense: 5’-ATTGCTTGGCTGGTGAAAGG-3’** |
|  |  | **anti-sense: 5’-TCGTCAGAGAAGGAGCCATC-3’** |
| **GluR** | **mouse** | **sense: 5’-TGTTCCTCAGCTCCTTCCAG-3’** |
|  |  | **anti-sense: 5’-AAGAGCTTTGCCTTCTTGCC-3’** |
| **INS1** | **mouse** | **sense: 5’-ACCCTGACAAGAGCAACTGA-3’** |
|  |  | **anti-sense: 5’-AGGTTCCTGTTGCTGTGACT-3’** |
| **IRS1** | **mouse** | **sense: 5’-CCTCCAACCTCCCTGAT-3’** |
|  |  | **anti-sense: 5’-TACACTCTTGGGGCTCATGG-3’** |
| **Leptin** | **mouse** | **sense: 5’-TGGAAGCCTCACTCTACTCCA-3’** |
|  |  | **anti-sense: 5’-ACATGATTCTTGGGAGCCTGG-3’** |
| **Leptin receptor** | **mouse** | **sense: 5’-CGTTCTGCAAATCCAGGTGT-3’** |
|  |  | **anti-sense: 5’-AGTCATCGGTTGTGTTCGGT-3’** |
| **LPL** | **mouse** | **sense: 5’-ATGGAGAGCAAAGCCCTG-3’** |
|  |  | **anti-sense: 5’-TCAGCCAGCCTTCTTCAGAG-3’** |
| **NPY** | **mouse** | **sense: 5’-CCTTCCATGTGGTGATGGGA-3’** |
|  |  | **anti-sense: 5’-GCAGACTGGTTTCAGGGGAT-3’** |
| **PPAR-γ** | **mouse** | **sense: 5’-CTGTGAGACCAACAGCCTGAC-3’** |
|  |  | **anti-sense: 5’-ATGGCATCTCTGTGTCAACCAT-3’** |
| **UCP1** | **mouse** | **sense: 5’-CGACTCAGTCCAAGAGTACTTCTCTTC- 3’** |
|  |  | **anti-sense: 5’-GCCGCCTGAGATCTTGTTTC-3’** |
| **18S** | **mouse** | **sense: 5’-TAACCCGTTGAACCCCATT-3’** |
|  |  | **anti-sense: 5’-CCATCCAATCGGTAGTAGCG-3’** |
| **GAPDH** | **mouse** | **sense: 5’-ACAGGGTGGTGGACCTCATGGT-3’** |
|  |  | **anti-sense: 5’-TGATGGTACACAAGGCAGGGCT-3’** |
| **PPAR-a** | **mouse** | **sense:  5’-TCCCTGTGAACTGACGTTTG-3’** |
|  |  | **anti-sense :  5’-AGTGGGGAGAGAGGACAGATG-3’** |
| **GLUT4** | **mouse** | **sense: 5’-ACGGATAGGGAGCAGAAC-3’** |
|  |  | **anti-sense: 5’-AAGGGTGAGTGAGGCATT-3’** |
